# Book Reviews

**Published:** 1893-12

**Authors:** 


### *BOOK REVIEWS.*

A Manual of Medical Treatment, or Clinical Therapeutics. By I. Burney Yeo, M. D., F. R. C. P., Professor of Clinical Therapeutics in King's College, London, and Physician to King's College Hospital, etc.

We have before us this work in two volumes from Lea Brothers & Co., Philadelphia. It is good, and we sincerely commend it to the physician and student. The illustrations are good, and the methods of treatment will commend themselves and offer suggestions of much value to the busy physician, and to those who are just beginning the work. C. S. W.

---

Hygiene and Public Health. By Lewis C. Parker, M. D., D. P. H., London University, Lecturer on Public Health at St. George's Hospital Medical School, etc. Third edition, 523 pages, with illustrations. Philadelphia, P. Blakiston, Son & Company, 1892.

The fact that a third edition of this work was necessary in two years bespeaks its value. It is the outcome of the experience of one of the best teachers in London, and the author's aim seems to have been attained, announced in the preface, "to occupy within a small space nearly the whole field of sanitary science," and enable the reader "to refer with advantage to the larger and more abstruse text-books." The work is concise, modern, not over-loaded and padded with statistical and other tables, simple and direct in style, and should be popular among students and instructors.

---

Leonard's Physician's Pocket Day-Book. Bound in red morocco, with flap, pocket and pencil loop. Price, postpaid, $1.00. Published annually by the Illustrated Medical Journal Co., Detroit, Mich.

This popular day-book is now in its sixteenth year of publication. It is good for *thirteen* months from the first of *any* month that it may be begun, and accommodates charges for fifty patients daily for that time, besides having cash department and complete obstetric records. There is space for the diagnosis of each case, or for brief records of the treatment adopted, following each name-space. Name of each patient needs to be written but three times in a month. It has the usual printed matter, such as: Dose List, Poisons and Antidotes, Urinary Tests, Exanthematicæ, Disinfectants, Weights and Measures. The book is seven and a half inches, long and three and a half inches wide, so that it will carry bill heads or currency bills with out folding. It is bound in flexible covers, and weighs but five ounces, so that it is easily carried in the pocket.

---

A Practical Treatise on Diseases of the Skin, for the use of students and practitioners. Third edition, revised and enlarged. By James Nevins Hyde, A. M., M. D., Professor of Skin and Venereal Diseases, Rush Medical College, Chicago, etc. Philadelphia, Lea Bros. & Co., 1893.

This work appears thoroughly up to date. Many new diseases of the skin are described, and additional matter added to chapters on older diseases. The author has thoroughly covered the ground of dermatology, beginning with an excellent description and cuts of the anatomy of the skin. The exanthemata are also very properly described.

Chancre is included in the description of the syphilodermata. As a rule dermatological authors omit it, and very wrongly.

The plates are especially good. The majority of the wood cuts are good, but the rest are poor. This is probably the most complete and thorough American work on dermatology. M. B. H.

---

The Principles and Practice of Surgery. By John Ashhurst, Jr., M. D., Professor of Surgery and Clinical Surgery in the University of Pennsylvania, Surgeon to the Pennsylvania Hospital, Senior Surgeon to the University Hospital, etc. Sixth Edition, Enlarged and Revised. Lea Bros. & Co., Philadelphia.

"Ashhurst's Surgery" has enjoyed a well deserved popularity among students and physicians for many years. In revising the work for the present edition, all the changes and improvements have been made which appeared to the author advisable from the additions that have been made to our surgical knowledge and technique. Dr. Ashhurst is a conservative teacher, and in his description of surgical measures very properly omits most of the later-day "fads" which have not stood the test of time. One new chapter, of great value, has been added, on Surgical Bacteriology, by Dr. Chas. B. Nancrede, of the University of Michigan. Portions of the work relating to gynecology have been revised by Dr. Barton C. Hirst, of Philadelphia. Many of the statistical tables contained in the former editions have been omitted. They were compiled from records so old as to have little value to-day.

We do not know of a more compact and practical work on surgery than this, and we are sure that the popularity which it has heretofore enjoyed will be bestowed upon the sixth edition.

